# Supplementary material for: Hyperparametric solitons in nondegenerate optical parametric oscillators
Source: Nat Commun. 2026 Feb 28;17:3329. doi: 10.1038/s41467-026-70122-x (PMC13066387; doi:10.1038/s41467-026-70122-x)
Supplement: Supplementary file 1 — Supplementary Information [file 41467_2026_70122_MOESM1_ESM.pdf]

Supplementary Information: Hyperparametric solitons in nondegenerate optical parametric oscillators

Haizhong Weng[1,2], Xinru Ji[3], Mugahid Ali[1], Edward H. Krock[1], Lulin Wang[1], Vikash Kumar[1], Weihua Guo[4], Qing Wan[2], Tobias J. Kippenberg[3], John F. Donegan[1,*] and Dmitry V. Skryabin[5,6,7,**]

[1] *School of Physics, CRANN, AMBER and CONNECT, Trinity College Dublin, D02 PN40, Dublin 2, Ireland*
[2] *Center for Heterogeneous Integration of Functional Materials and Devices, Yongjiang Laboratory, 315202, Ningbo, China*
[3] *Institute of Physics, Swiss Federal Institute of Technology Lausanne (EPFL), CH-1015 Lausanne, Switzerland*
[4] *Wuhan National Laboratory for Optoelectronics, and School of Optical and Electronic Information, Huazhong University of Science and Technology, 430074, Wuhan, China*
[5] *Department of Physics, University of Bath, Bath, BA2 7AY, United Kingdom*
[6] *Centre for Photonics, University of Bath, Bath, BA2 7AY, United Kingdom*
[7] *National Physical Laboratory, Teddington, TW11 0LW, United Kingdom*
[*] *Corresponding author: jdonegan@tcd.ie*
[**] *Corresponding author: d.v.skryabin@bath.ac.uk*

SUPPLEMENTARY NOTE 1. MICRORESONATOR DESIGN AND OPO OPERATION

The initial design objective was to achieve phase matching for the fundamental transverse magnetic mode (TM_{00}) for generating far-detuned signal and idler fields by optimizing the ring width (RW) and ring thickness (RT), while keeping the outer radius at 114.38 μm (FSR$\approx$200 GHz) and the pump frequency at approximately 191.5 THz ($\mu = 0$), respectively. Supplementary Figures **1a** and **1b** show the simulated frequency mismatch and group velocity dispersion (GVD) as a function of RT, with a fixed RW of 2500 nm. When RT goes above 750 nm, the pump mode enters the anomalous dispersion regime, and the phase-matched signal and idler frequencies appear across the broad range of frequencies controlled by the waveguide width: 220.4 THz to 265.2 THz interval for the signal and 162.6 THz to 117.6 THz for the idler for RT between 750 nm and 810 nm. Throughout this range, the signal and idler modes remain in the anomalous and normal dispersion ranges, respectively.

Supplementary Figure **1c** shows that increasing RW from 2000 to 3000 nm, while keeping RT constant at 770 nm, decreases the frequency separation between the signal and idler waves. To target signal and idler modes near the O-band and 2 μm wavelength regimes, respectively, we scanned the RW values around 2500 nm. Supplementary Figure **1d** shows the experimental transmission spectra of a resonator with a cross-section 770 nm$\times$2520 nm, coupled to a pulley waveguide with a 650 nm gap and a coupling angle of 25°, see Figs. **2a** and **2b**. The TM_{00} modes exhibit strong coupling rates in the C-band, as plotted in the top panel. This is verified by their broad linewidth and low extinction ratio, in contrast to the first-order modes (TM_{10}), which exhibit weaker coupling. In the O-band (lower panel), the extinction ratios of the TM_{00} modes are relatively high, indicating weaker coupling and higher loaded quality factors, as shown in Fig. **2d**.

The Supplementary Figure **2a** shows phase-matching plots for the varying pump mode numbers and a given resonator geometry. A zoomed-in view of the frequency mismatch is provided in the left inset, where diamonds represent resonant positions, and circles indicate the points of minimum mismatch. Supplementary Figure **2b** shows experimental results that are consistent with the phase-matching analysis. When the pump mode shifts by one FSR (200 GHz), the idler and signal shift by one to four FSRs, depending on which signal-idler pair provides best phase-matching for the shifted pump. Generally, powers of the signal and idler waves decrease as the generated sidebands move further from the pump mode. Supplementary Table 1 shows computed normalised phase-matching and linewidth parameters for $\pm\mu$ signal-idler pairs used in numerical modelling discussed in the Methods section.

Supplementary Table I. Normalised phase-matching and linewidth parameters for $\pm\mu$ signal-idler pairs.

μ	250	251	252	253	254	255	256
$\Delta f_\mu/\kappa_0$	6.776	5.442	4.078	2.683	1.258	-0.198	-1.686
κ_μ/κ_0	0.2785	0.2760	0.2734	0.2709	0.2685	0.2660	0.2636
μ	-250	-251	-252	-253	-254	-255	-256
κ_μ/κ_0	0.3281	0.3388	0.3496	0.3607	0.3719	0.3833	0.3949

I. SUPPLEMENTARY NOTE 2. EXPERIMENTALLY OBSERVED DYNAMICS OF HYPERPARAMETRIC SOLITONS

The Supplementary Video file (ndOPO_soliton.mp4) captures the evolution of the microcomb spectra, encompassing both the signal and pump wavelength regions, as the laser wavelength is manually increased with a constant on-chip pump power of 425 mW. Watching this footage, one can see how the system progresses through a series of dynamic nonlinear states and eventually enters the soliton regime. The initially excited signal line gradually intensifies and forms a frequency comb (time

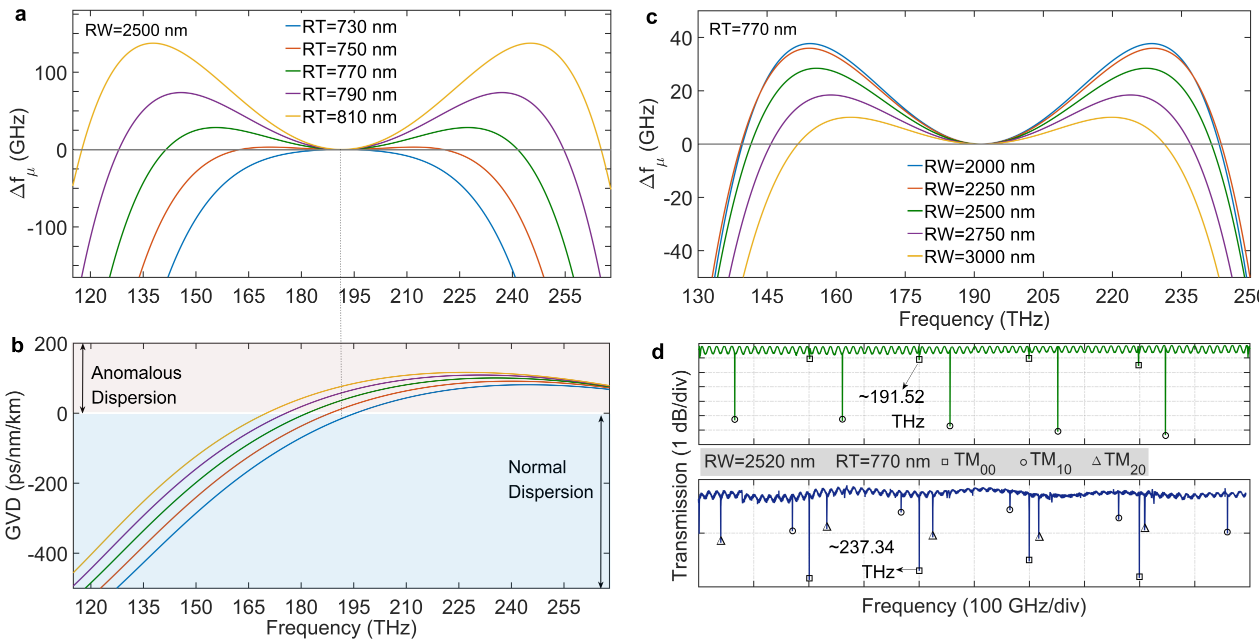

Supplementary Fig. 1. **Microresonator properties.** **a**. Simulated frequency mismatch and **b** group dispersion (GVD) properties at various ring thickness (RT) with a fixed ring width (RW) of 2500 nm. **c**. Simulated frequency mismatch as a function of RW variation, with the RT fixed at 770 nm. **d**. Experimental TM-polarized transmission spectra, for a resonator with a dimension of 770×2520 nm, in C-band (upper) and O-band (lower), respectively. The squares, circles, and triangles indicate the TM_{00}, TM_{10}, and TM_{20} modes, respectively.

0 : 12), followed by the emergence of hyperparametric soliton crystals and multi-soliton states (time 0 : 18). One can also see how the contrast in power between the signal central line and the powers of the surrounding comb lines is reduced when soliton crystals are generated. As the laser frequency continues to change, various multi-solitons are observed between times 0 : 43 and 1 : 26. During the manual tuning process, soliton states exhibit variability due to drifts of the instantaneous pump parameters. However, once the laser wavelength is held constant, the resulting soliton state becomes stable.

II. SUPPLEMENTARY NOTE 3. REPETITION RATE CHARACTERISATION OF THE HYPERPARAMETRIC SINGLE-SOLITON

The coherence of hyperparametric OPO solitons was verified through radio frequency (RF) noise measurements by heterodyning a single microcomb line with an auxiliary C-band tuneable semiconductor laser (TSL), see main text and Methods. In addition, we characterised the soliton repetition rate (f_{rep}) using electro-optic (EO) modulation driven by an RF signal. As illustrated in Supplementary Fig. **3a**, the generated OPO soliton was injected into a thin-film lithium niobate-based EO comb generator developed by Liobate Tec. [1]. This EO comb generator consists of a cascaded on-chip intensity modulator and a phase modulator, both of which are driven by a common microwave source at $f_{mod} = 25$ GHz. To improve the modulation depth, an RF amplifier was used specifically for the phase modulator, enabling the generation of higher-order sidebands that overlap with adjacent Kerr comb lines. Subsequently, an optical band-pass filter (BPF) was used to isolate comb lines around 191.2 THz (corresponding to $\mu = -1$) for beat note analysis. The schematic diagram in Supplementary Fig. **3b** illustrates the relationship between the Kerr soliton comb lines (thick blue) and EO-generated sidebands (thin red). The solid and dashed red lines represent EO sidebands originating from the $\mu = 0$ and $\mu = -1$ modes, respectively, with the frequency offset Δf, while the soliton comb repetition rate is evaluated as $f_{rep} = 8 \times f_{mod} - \Delta f$.

III. SUPPLEMENTARY NOTE 4. TUNABILITY OF THE HYPERPARAMETRIC SOLITONS

Phase-matching plots in Supplementary Figs. **1a** and **1c** show that the signal-idler frequencies can be tuned in the cw-regime by varying the resonator waveguide dimensions. By fixing the pump mode near 191.1 THz, we pumped resonators with RW of 2530 nm, 2570 nm, and 2610 nm, with high power to demonstrate tunability of the soliton states, see Supplementary Fig. **4a**. We indeed demonstrated generation of the hyperparametric soliton crystals with distinct signal frequencies of 240.3 THz, 239.2 THz, 238 THz, respectively, following a trend similar to that shown in Supplementary Fig. **1c** for the

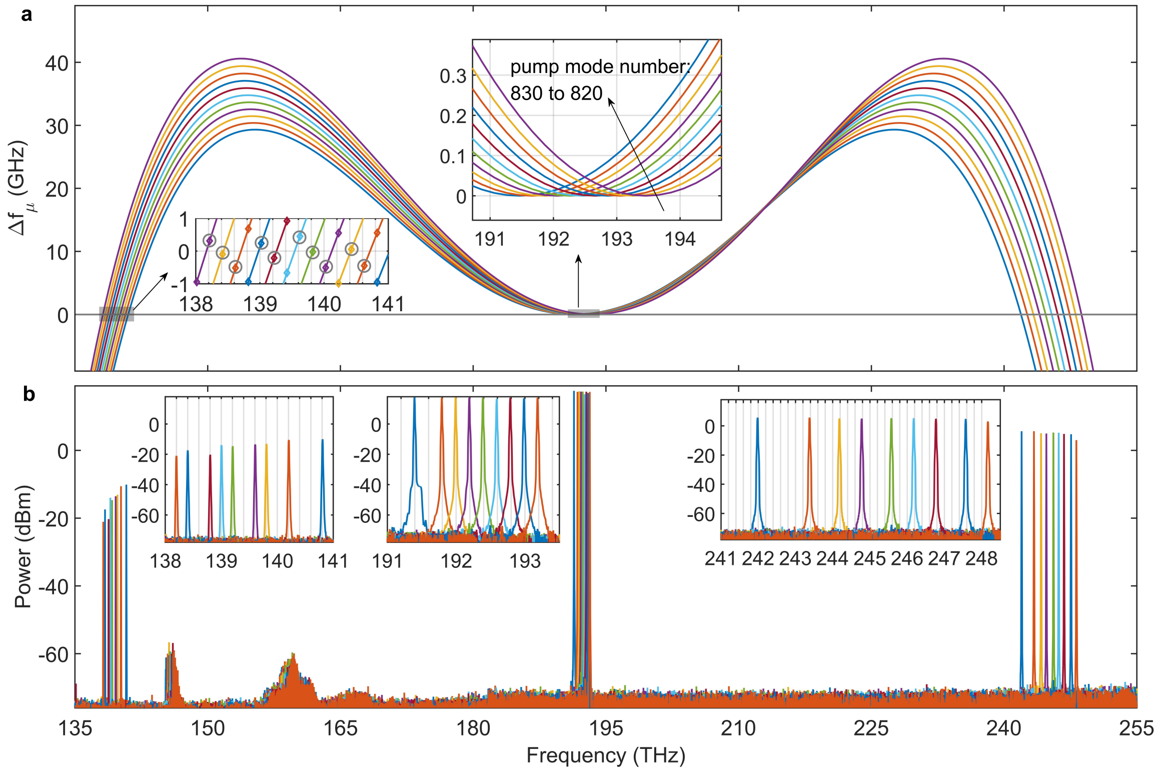

Supplementary Fig. 2. **Frequency mismatch and low-power OPO spectra.** **a**. Simulated frequency mismatch properties for the target resonator (770 nm×2520 nm) with different center modes. Insets: zoomed-in views of the mismatch near the pump and idler frequencies, where diamonds and circles indicate the frequency mismatch at resonances and near-zero values, respectively. The 200-GHz (one FSR) grid lines are used for reference. **b**. Measured OPO excitation spectra when pumping various resonant TM_{00} modes with a constant on-chip power of 250 mW. Insets: zoomed-in views of the spectra for the three waves, with 200-GHz grid lines.

low power CW-regime. To explore how different pump modes affect soliton formation, we examined the comb generation by pumping a series of distinct TM_{00} modes using the same resonator as in the main text (RW= 2520 nm), see Supplementary Fig. **4b**. Specifically, for the pump frequencies 191.4 THz, 192.4 THz, 193.0 THz, and 193.6 THz, we observe the generation of hyperparametric single-solitons centred at 242 THz, 245.4 THz, 247.4 THz, and 249.4 THz, respectively.

While most of the solitons in this study are generated at room temperature, we find that a moderate change (e.g., within $\sim 30\,^{\circ}$C) in the chip temperature, induced by a thermoelectric cooler on the substrate, expands the number of accessible resonances that support soliton formation, enhancing the versatility of the soliton states. The different noise levels between Supplementary Figs. **4a** and **4b** arise from the use of different OSAs. Throughout all experiments, the on-chip pump power required to access soliton states lies in the range of $400 - 500$ mW. Higher power levels lead to non-solitonic combs, see next section and Supplementary Fig. **5**.

The solitons we reported were readily accessed by tuning the pump laser into resonance at a relatively slow rate, without the need for active thermal control, supporting our conclusion that they are generated at the spectral tail of the resonance, where intraresonator cw power buildup and thermal effects are not important. Overall, our observations confirmed the robustness and tunability of hyperparametric solitons in nondegenerate OPOs. The tunability aspect enables the engineering of such OPOs for applications in coherent optical communications and spectroscopy.

IV. SUPPLEMENTARY NOTE 5. NON-SOLITONIC FREQUENCY COMBS

Now, we present the experimental results for the relatively high on-chip pump power of 550 mW, when the hyperparametric solitons are no longer observed. The corresponding comb spectra recorded at different detunings are shown in Supplementary Fig. **5**a. For large detunings, the power of the generated comb lines increases gradually, but then it jumps up, signalling a transition in the shapes of the measured spectra, see Supplementary Fig. **5a**. The initial stage (spectra (i-iv)) is characterised by the primary three-colour OPO combs separated by 11×FSRs. The 11-FSR spectral gap is gradually filled by the secondary comb lines [3] as we tune the pump frequency.

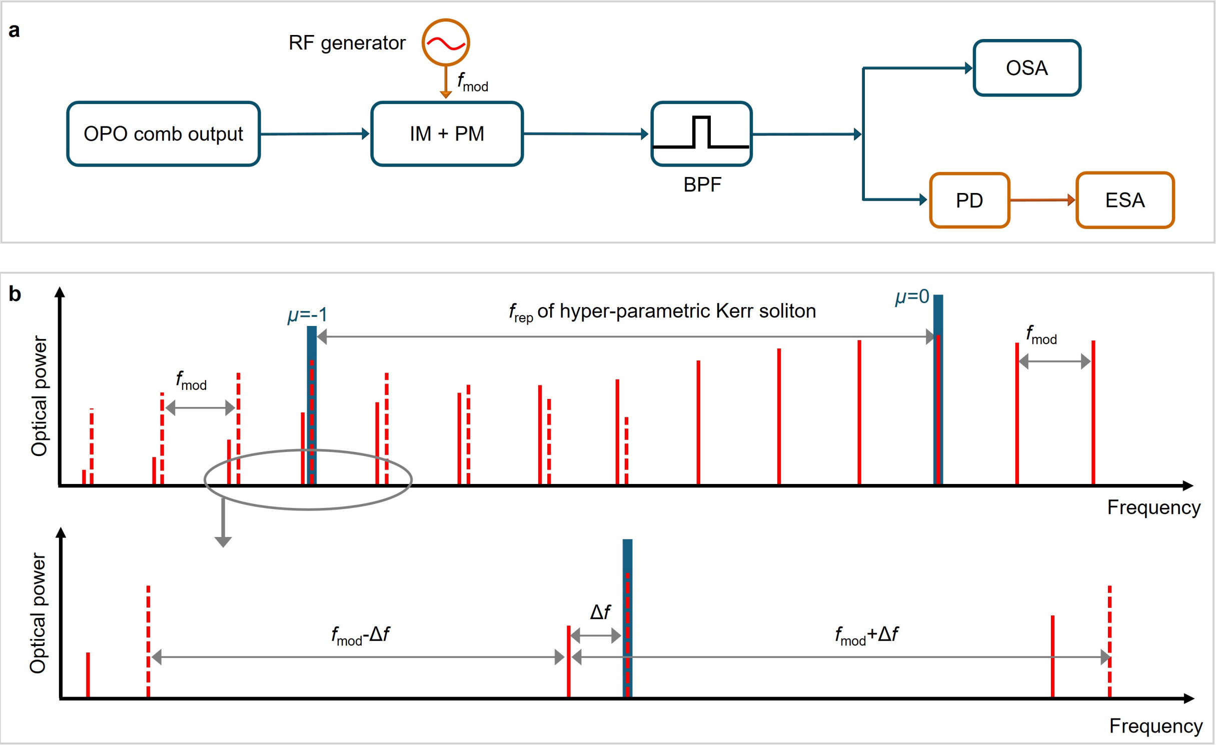

Supplementary Fig. 3. **Repetition rate measurements concept. a**. Experimental setup for measuring the repetition rate of the hyperparametric single-soliton. IM: intensity modulator; PM: phase modulator; BPF: band-pass filter; OSA: optical spectrum analyzer; PD: photodiode; ESA: electrical spectrum analyzer. **b**. Schematic diagram illustrating the spectrum after modulation, corresponding to the Fig. **3e**.

For the spectra in the panel (v), this structure is replaced by a dense spectrally homogeneous incoherent comb, which finally transitions to the state (vi) when the pump begins generating its own Kerr-only comb (with primary comb lines separated by 28×FSRs), which does not translate to the signal and idler combs. This behaviour can be attributed to the saturation of nonlinear conversion from the pump to the signal and idler, leaving residual pump power available to drive the non-OPO comb around the pump. The green inset in the panel (vi) shows a zoomed-in view of the spectrum near the first from the pump Kerr comb line at 186 THz. Notably, the new Kerr comb is much stronger than the OPO one, explaining the sharp increase in the power seen in the top inset. The corresponding low-frequency noise measurements confirm that the combs in this series of measurements are incoherent, apart from the very first one representing a coherent three-colour Turing pattern state, see Supplementary Fig. **5b**.

[1] J. Wang, Q. Wang, M. Xu, Y. Zhu, Y. Wang, H. Deng, and X. Cai, Highly tunable flat-top thin-film lithium niobate electro-optic frequency comb generator with 148 comb lines, Optics Express **33**, 23431 (2025).

[2] S. Zhang, J. M. Silver, T. Bi, and P. Del'Haye, Spectral extension and synchronization of microcombs in a single microresonator, Nature Communications **11**, 6384 (2020).

[3] T. Herr, K. Hartinger, J. Riemensberger, et al. Universal formation dynamics and noise of Kerr-frequency combs in microresonators. Nature Photonics **6**, 480–487 (2012).

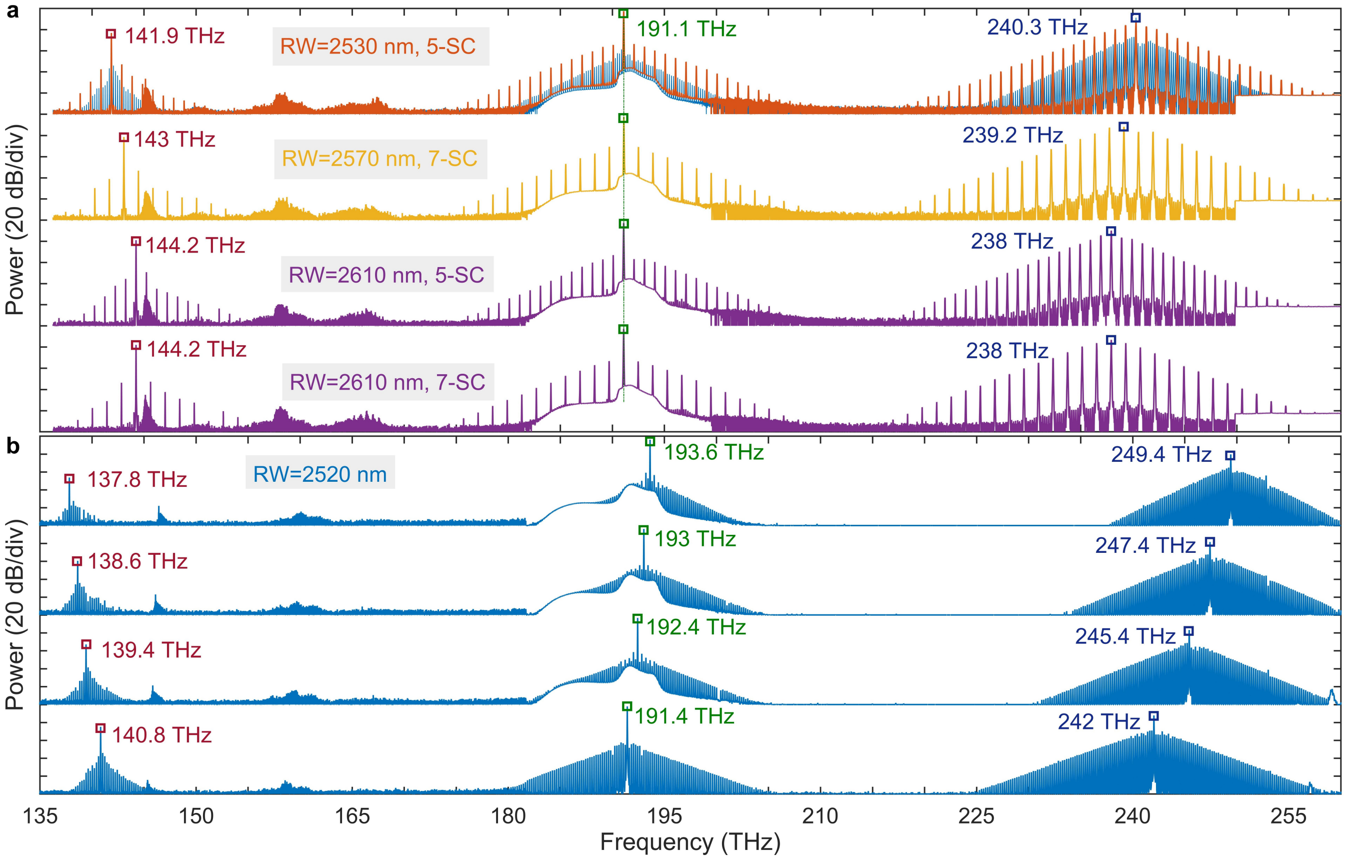

Supplementary Fig. 4. **Spectral tuning of solitons by changing resonator geometry and pump frequency. a**. Hyperparametric soliton crystals generation with varied signal and idler frequencies, achieved with the nearly same pump frequency in three resonators with different RWs. **b**. Hyperparametric single-soliton generation while tuning the pump frequency across different TM_{00} modes within the same resonator.

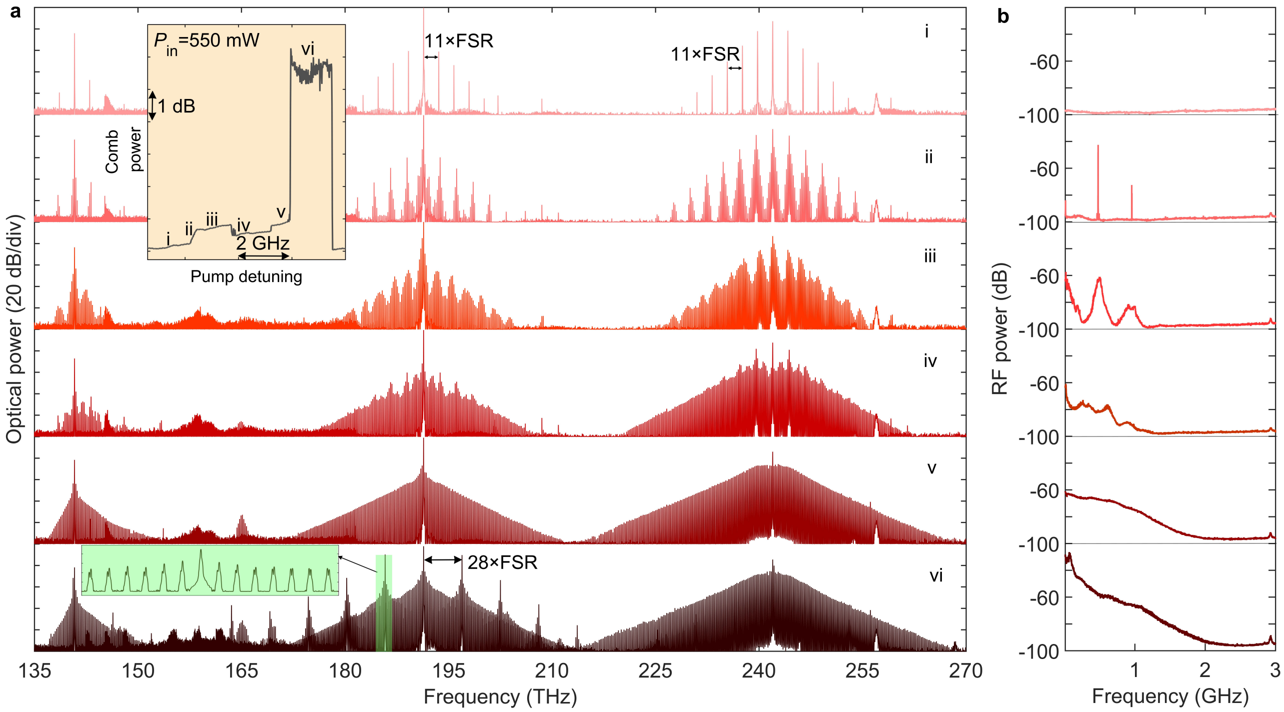

Supplementary Fig. 5. **Broadband OPO spectra for varying pump frequency.** **a**. Non-solitonic combs recorded at 550mW of pump, with varying pump detunings as indicated in the inset. The resonator used is the same as in the main text (RW= 2520 nm). **b**. Corresponding RF noise measurements.
