# Supplementary material for: Hyperparametric solitons in nondegenerate optical parametric oscillators
Source: Nat Commun. 2026 Feb 28;17:3329. doi: 10.1038/s41467-026-70122-x (PMC13066387; doi:10.1038/s41467-026-70122-x)
Supplement: Supplementary file 2 — Description of Additional Supplementary Files [file 41467_2026_70122_MOESM2_ESM.pdf]

Description of Additional Supplementary Files

Supplementary Video 1. Video showing how the signal and pump spectra evolve during manual frequency tuning across the hyperparametric soliton range. Initially, non-solitonic combs replace the monochromatic signal and pump, which then evolve into soliton generation. The strong, 8dBm, central sideband in the signal component rising above the comb starting around -8 dBm, unambiguously points to the hyperparametric regime. The soliton existence on the negatively detuned tail of the resonance, where intraresonator fields and thermal effects are modest, enables the simplicity of the manual tuning. Tuning to the range of positive detunings boosts the circulating power, leading to the sudden loss of resonance due to thermal shifts.
